# HIV in MOTION: a community of practice on physical rehabilitation for and by people living with HIV and their allies

**DOI:** 10.3389/fresc.2023.1154692

**Published:** 2023-10-06

**Authors:** Francisco Ibáñez-Carrasco, Tizneem Jiancaro, Brittany Torres, Kiera McDuff, George Da Silva, Joanne Lindsay, Colleen Price, Shaz Islam, Glen Bradford, Kelly K. O'Brien

**Affiliations:** ^1^ Dalla Lana School of Public Health, University of Toronto, Toronto, ON, Canada; ^2^ Department of Physical Therapy, Temerty Faculty of Medicine, University of Toronto, Toronto, ON, Canada; ^3^ MAP Centre for Urban Health Solutions, Unity Health, St. Michael's Hospital, Toronto, ON, Canada; ^4^ AIDS Vancouver, Vancouver, BC, Canada; ^5^ Rehabilitation Sciences Institute, University of Toronto, Toronto, ON, Canada

**Keywords:** disability, rehabilitation, HIV inclusion, community of practice, evaluation, exercise, physical rehabilitation

## Abstract

**Background:**

This paper describes the design, implementation, and evaluation of a community of practice (CoP), HIV in MOTION (HIM), to advance physical activity rehabilitation interventions with adults living with HIV, clinicians, researchers, and representatives from community-based organizations. We attracted a diverse audience of geographically dispersed people living with HIV, clinicians, exercise personnel, and trainees to eight HIM community of practice events that featured the clinical, research, and lived experience of people living with HIV. HIV in MOTION had (a) a domain related to physical rehabilitation, exercise, and social participation for people living with HIV; (b) a community of diverse individuals; and (c) a practice, that is, a series of sustained interactions online and offline, synchronous, and asynchronous. Our team included six diverse people living with HIV, two coordinators, and three academic researchers who planned, prepared, implemented, and evaluated each online session. To evaluate the HIV in MOTION CoP, we employed an evaluation framework composed of five criteria: Goals and Scope, Context and Structure, Process and Activities, Outcomes, and Impact. We collected quantitative and qualitative evaluative data using online evaluation, audiovisual archiving, and participant observations during the debriefing with all members of our team.

**Results:**

We widened the Goals and Scope of the HIV in MOTION CoP to include the HIV narrative of lived experiences, including autopathography, and participant storytelling. In matters of Context and Structure, we received explicit satisfaction with our governance and leadership. Also, being flexible to fit online formats was a productive strategy that made the HIV in MOTION CoP sessions agile and amenable to audiovisual archiving. Our indicators of success in Process, Activities, and Outcomes included participant retention online, elicited verbal interventions and comments in the chat room, and a rate of three repeat visits online. The indicators of success of Impact were the presence of voluntary and unscripted autopathography, the patient storytelling and how it reportedly caused changes in the participants, and the “legitimate peripheral participation” of emerging research and clinical students. In conclusion, we recommend our form of CoP for mixing the knowledge of diverse persons in this area. However, we recommend considering budget and burnout as serious challenges to sustainability.

## Introduction

*Thinking together about real-life problems that people genuinely care about gives life to Community of Practices (CoPs)* (1).

Succinctly described, a community of practice (CoP) is a group of people who “share a concern or a passion for something they do and learn how to do it better as they interact regularly” (2). The concept was first proposed by cognitive anthropologist Jean Lave and educational theorist Etienne Wenger in their germinal 1991 book, “Situated Learning,” and applied and expanded since then (3, 4).

Working on the proven premise that CoPs mobilize research evidence (5), we set out to mobilize evidence of the role and impact of exercise and physical activity among adults living with HIV. We wanted to create opportunities for partnerships, collaboration, and information sharing. The main purpose of our CoP was to contribute to bridging gaps between research evidence and practice in physical rehabilitation and social participation for people living with HIV.

In this paper, we describe the design, implementation, and evaluation of a sustainable community of practice called HIV in MOTION (HIM) to advance physical activity rehabilitation interventions with adults living with HIV, clinicians, researchers, and representatives from community-based organizations (https://rise.articulate.com/share/HgA3hWAtWNqoIR8iKkfzdsAU0cLknrmY#/). HIV in MOTION was part of a community-based research study (2019–2023) to develop and assess the utility of a short-form HIV-specific disability patient-reported outcome (PRO) questionnaire, a tool to identify the presence, severity, and episodic nature of disability experienced among adults living with HIV for use in community-based settings, which include AIDS Service Organizations (ASOs), community health centers, and clinics.

The HIV in MOTION CoP (https://hivinmotion.ca/) and the accompanying study are inscribed in a context where people who are HIV positive often experience more additional health problems and aspects of disability compared to those who do not have HIV, especially as they age. This is worsened by layers of uncertainty about the future and perceived, internalized, and/or enacted forms of stigma (e.g., for having acquired HIV through sex or use of substances) (6). The effectiveness of exercise for people living with HIV has been stated in systematic reviews including the one by O’Brien et al. (7), a Framework of the Physical Therapy Role in HIV Care in 2019 (8), and collaborative research priorities (9). In addition, research evidence tells us that rehabilitation services and programs are sorely needed by many of the estimated 62,790 people were living with HIV in Canada (People living with HIV in Canada: infographic, Accessed August 16, 2023) especially by those over 50 years who make nearly 50% of this population (10–12).

Research has found statistically significant benefits of aerobic and resistive exercise among people living with HIV. Also, various forms of movement and exercise have been found to support their cognition and increase social participation (7, 13). However, the engagement and uptake of exercise among adults living with HIV varies a great deal (14). In Canada, these foundational research findings show that there are several things we need to do. One of these important things is to teach healthcare workers, people who work in non-profits, and individuals with HIV about how exercise and movement are helpful for people with HIV. This is especially important for those who are getting older while living with HIV. This idea led to the creation of an online community called the “HIV in MOTION Community of Practice.”

### Communities of practice online and knowledge transfer

When we started the CoP, we wanted to attract a diverse audience of geographically dispersed people living with HIV, clinicians (occupational therapists (OTs), physical therapists (PTs), general practitioners, HIV medicine), exercise personnel, and trainees to events that featured prominently not only the clinical and research aspects but also the “lived experience” of people living with HIV.^1^

We stayed away from traditional expositive formats such as lectures that favor the academic voices and knowledge. We took advantage of the social aspects of what has been called the “Social Web” because its content can be easily generated and published by users, and “the collective intelligence of users encourages more democratic use” (16). This *democratic* aspect is key to understanding the flexible, upbeat, informative, and “low-complexity” (17) format of our CoP. We developed the HIV in MOTION CoP to support the growing interest and practice of social and physical activity among people living with HIV of all ages, no matter how challenged.

CoPs are a form of knowledge transfer (also referred to as integrated knowledge mobilization or iKMb). Specifically, integrated knowledge transfer (iKT) is an approach that applies community-based research principles and practices, such as co-ownership, collaboration, co-production of knowledge, and balancing the differing community–academic–clinical powers (e.g., perceived authority of the medical discourse about the patient) (18–22). iKT is “an alternative approach for promoting research use in which research users function as active partners to generate research from conceptualisation to implementation, rather than passive recipients of research or research products” (18, 23).

Research in this area reports that virtual CoPs are forms of active and collaborative co-learning about a shared concern, collective learning in regular interactions, identity-building, and knowledge mobilization (1, 3, 24–26). CoPs have been found to promote benefits related to patient diagnosis and treatment, clinical management, trust across sectors, supports for HIV healthcare providers initiating their practice, and updates on HIV healthcare and social services providers (27–29). The participation in a CoP can range from one-offs to more constant participation in a core group. Of note, it has been said that “[the involvement of] community is a facilitator of the knowledge sharing in CoPs” regardless of the frequency of their participation (30). CoPs give healthcare professionals “a stronger collective sense of their roles” (31) by creating “professional awareness regarding patient empowerment” (32).

CoPs share a basic structure of three elements: (a) a *domain* or topics that drive the community, that is, a range of matters related to physical rehabilitation, exercise, and social participation for people living with HIV; (b) a *community* of diverse individuals who recognize and value each other and the core subject matters; and (c) a *practice*, that is, a series of sustained interactions online and offline, synchronous, and asynchronous (25). In the next section, we describe how these three elements and forms of participation interplayed in the design and implementation of the CoP.

### Designing and implementing the HIV in MOTION CoP

Following CoP examples in the field, our design was flexible and participatory (33) and informed by our team's prior experience with online learning related to social–behavioral and physical aspects of living with HIV since 2009 (34, 35). We also tapped into an existing international collaborative center led by one of our academic authors (https://cihrrc.ca). We designed and implemented HIV in MOTION online sessions four times per year in collaboration with a group of six HIV+ “Ambassadors.” The goal of each CoP session was to increase knowledge about exercise and physical activity in the context of HIV and to foster dialog, collaboration, and support in the field. The Ambassadors participated in the choice of topics, identifying potential speakers, etc., alongside the research leads and two coordinators, one Research Coordinator, and one Engagement Coordinator living with HIV. The latter's role was to support the Ambassadors and all persons living with HIV in the accompanying studies. Ambassadors were provided a yearly honorarium of 1,000 Canadian dollars (CAD) for their involvement in HIV in MOTION.

Our design emphasized the value of being informed and designed by “patients” directly. Our ambassadors have long-standing connections to AIDS service organizations and with communities impacted by HIV, and they included cis-men and women, trans persons, and HIV+ staff of regional HIV non-profits. Many of the Ambassadors had prior experience as *peer researchers* who are patients that participate in any or all the people living with HIV of a research project over time (36). We relied on a framework to support ongoing effort at engagement and opportunities to participate in panels, conference posters, and events (36).

**Figure 1:**
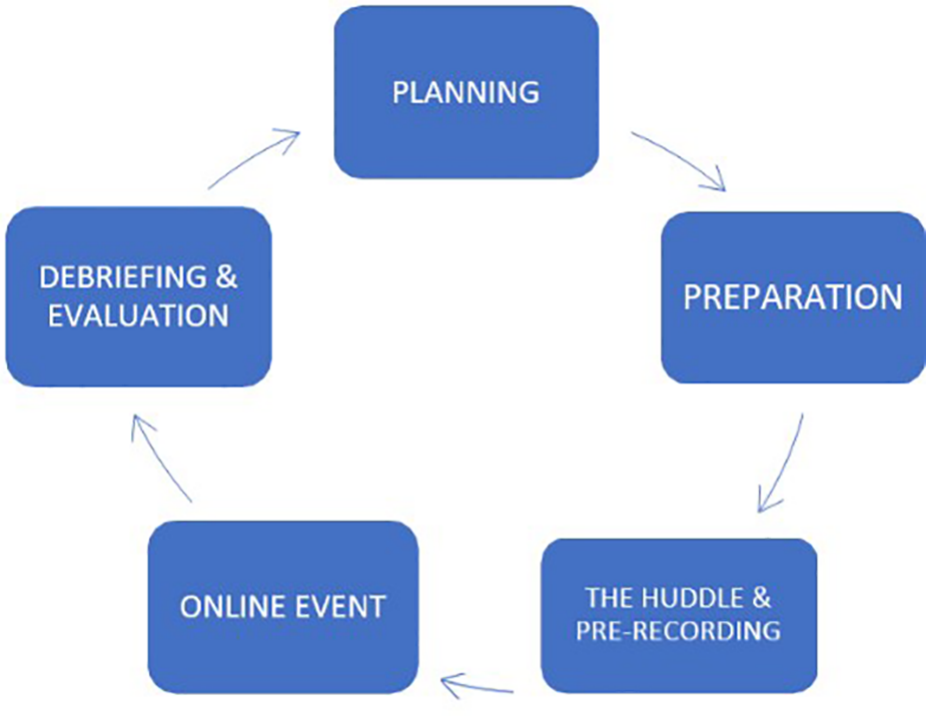
Steps to design, implement, and evaluate the HIM CoP.

The process of designing and implementing each HIV in MOTION online event encompassed five steps (see Figure 1).

Step 1—Planning: we met quarterly with the Ambassadors, coordinators, and lead researchers to choose topics, speakers, and live demonstrations.

Step 2—Preparation: speakers, panelists, and those providing 5–10 min of physical activity demonstration were briefed about their involvement in an upcoming event. We sent useful tips for speaking to the webcam, the lights, and sound. We also provided a media release form. In this step, we also prepared and launched social media promotions and pre-recorded edited videos.

Step 3—The Huddle and pre-recording: days before the public CoP online event, the team, guest speakers, and panelists huddled online for 1 h to become acquainted with each other, offer an overview of the presentation, and receive technical support (e.g., lighting, etc., for live Zoom sessions).

Also, in Step 3, we recorded short sections to be both broadcasted and integrated into our video library (https://bit.ly/HIV-in_MOTION_Audiovisual_Library). For speakers, we offered 20–30 min. We offered 10–15 min to the guests providing physical demonstration (e.g., stretching while sitting).

We sent the panelists in advance a few guiding questions and the main speaker's pre-recorded video to give everyone an opportunity to reflect on the forthcoming content presented live online.

Nonetheless, we did not confine their responses to the perspectives the speakers were addressing.

Step 4—The online event: we broadcasted always at 12–2 PM EST with the aim of capturing as wide an English-speaking audience as possible. At all steps, we considered the differing time zones of speakers/panelists as well as audience members. Our records indicate ample participation from Canada, USA, UK, New Zealand, and Australia, and a few audience members from South Africa and Israel. We capped each session at 2 h or less. We alternated between two equally strong styles of event facilitation: one facilitator worked with prepared questions and a set order of interventions for panelists; the other facilitator used a conversational style akin to a talk show. The format most often included a welcome (5 min), followed by the pre-recorded exercise or mindfulness demonstration (10–15 min), and a pre-recorded speaker's presentation. Next, we broadcasted live (synchronous) one 40–60 min panel of up to five people, always including at least two persons with lived experience, the invited speaker and the invited exercise demonstrator (e.g., a physiotherapist, an HIV+ yoga practitioner, a local YMCA coach affiliated with our studies, etc.). The facilitator would gage the interest and timing and open the panel to all in the virtual room. A facilitator's sidekick would mind the chat room questions, curate them for repetition and how they would enhance the dialog, and pass some of them onto the facilitator or resolve them in the chat room.

Step 5—Debriefing and evaluation: the Team, Ambassadors and core research team members, met within 3 weeks after each CoP session to debrief. We checked on what worked and what did not, who attended, and what we should modify in the next iteration. We completed this process eight times for each CoP session. We emailed thank you notes to the participants along with an electronic gift card as a token of appreciation to speakers, panelists, and exercise demonstrators for their involvement in the CoP session. We sent all registrants links to the audiovisual library and a link to a digital evaluation. Speakers and panelists were given a modest honorarium.

### Evaluating HIV in MOTION

There is little agreement on approaches to evaluating the many aspects of CoPs (25, 37). In current scholarly work, we often measure the success of knowledge transfer activities by quantifying the number of events, participants, evaluations received, etc. (38). These standards are incommensurate with the small scale of the CoP, which produced eight events in 2 years. For an online influencer, this is peanuts. For a diverse community–academic team, this is a big effort. Literature in this regard suggests that CoPs enable micro-sociological, collaborative, and peripheral forms of participation that are not easily quantifiable (39).

To evaluate the CoP, we have employed one adapted evaluation framework composed of five criteria adapted from Alcalde-Rabanal et al. (40), McKellar (25), and Moore et al. (41). These criteria are amenable to be measured in quantitative and qualitative ways, and they include (1) *Goals and Scope*, (2) *Context* and *Structure* (e.g., types of participants and type of governance of network), (3) *Process* and *Activities* (e.g., outputs that evidence a degree of collaboration), (4) *Outcomes* (results from evaluation forms, audiovisuals produced), and (5) *Impact* (what may have changed as a result of our work in the CoP). These criteria allowed us to pay attention to motivation and participation, sprouting relationships, collective learning, and the formation or support of identities (37) as well as the nonlinearity, emergence, adaptation, uncertainty, dynamic interactions, and co-evolution of the CoP (42).

### Methodology of evaluation

We included three ways of collecting quantitative and qualitative data described in this section.

Online evaluation questionnaire: Among all eight sessions, 67 attendees completed the evaluation. After each CoP session, we administered an electronic self-reported evaluation questionnaire to all registrants and speakers using a link to the questionnaire.^2^ The questionnaire was composed of categorical response options (e.g., “Did you have a chance to connect with someone related to the HIV in MOTION Community of Practice, before, during or after the event?”) and open-ended questions to assess changes in knowledge, value, and connections made through the CoP planning, implementation, and post-CoP (see Supplemental File 1): “What were your overall thoughts on the HIV in Motion Community of Practice?” (Satisfaction and motivation), and “In what ways might you use the information shared at the HIV in Motion Community of Practice in your life or work?” (intent to use elements of the CoP).

The evaluation questionnaire included two retrospective pre- and post-measures of knowledge ranging from awareness to learning, for example, “Please rate on the scale from 1 (low level of knowledge) to 10 (high level of knowledge) your understanding/knowledge of [topic of the event] *before* the HIV in MOTION session of [date],” and a sister question about their knowledge *after* the event. To increase the response to the questionnaire, we raffled an e-gift card of 50 CAD to respondents, a strategy found not to influence the results (45).

Audiovisual archiving: literature in the area tells us that “audiovisual archiving” gathers outcomes and also promotes a long-term impact (46, 47). Ideally, such audiovisual materials are used by other researchers or in course instruction. More than 40 individuals have contributed to preparing the audiovisual materials of the CoP, and we hope that they have also contributed by using these materials to teach or train research teams (a long-term outcome not measured in this paper). Our audiovisual library operates, still today, as a free public asynchronous repository of our CoP efforts. To date, the library with podcasts and videos has received ≥1,500  hits over 2 years.

Recording, editing, seeking final approval from speakers, and formatting and classifying the material is a significant creative, budgetary, time, and energy effort. We paid a modicum for some of this work. The library supports the long-term goal of offering current information about physical rehabilitation, mental health benefits from exercising, and the lived experience of persons living with HIV. It has 19 short videos and podcasts (https://bit.ly/HIV-in_MOTION_Audiovisual_Library). This collection is enhanced by audiovisual work done prior to starting the CoP, also intended to highlight the research on, and the lived experience of, physical rehabilitation among people living with HIV. The collection includes presentations on sleep architecture, nutrition, and gender aspects of living with HIV. The collection also includes materials of a sister research project on conducting online exercise tele-coaching with persons living with HIV (48).

Participant observations: We relied on the participant observation of our Ambassadors and coordinators to put in perspective the resulting mix of formal presentations and the elicited conversation online during the event. We recorded the full session to review the presentation and comments. We told our audience that we would never make a public edited or unedited video without individual consent after each person had seen the edited clip.

Review of ethics: Considering the focus of this manuscript is centered around the delineation of the creation, execution, and assessment of a CoP, it is imperative to note that no ethical endorsement was sought for this endeavor. The CoP, in its essence, serves as a conduit for knowledge dissemination, and the individuals engaging in the CoP sessions and affiliating with its membership do not fall within the categorization of research subjects, but rather constitute integral components of a designated community of practice.

## Results

In this section, we provide results in each of the measured areas: (1) Goals and Scope, (2) Context and Structure, (3) Process and Activities, (4) Outcomes, and (5) Impact. To measure each area, we identified indicators of success in each area (see Table 1).

**Table 1:** HIM CoP evaluation criteria and identified indicators of success.

Criteria	Goals/scope	Context/structure	Process/activities	Outcomes	Impact
Quantitative indicators of success	No. of affiliations of all diverse participants (e.g., clinicians, patients, etc.)	No. of agreements and disagreements with governance and structure	No. of activities	No. of liaisons reportedly started as outcome of participating in CoP	Not measured
Qualitative indicators of success	Diverse participants report understanding the goal of the events	Sense of collective ownership Ease of access	Design of the prep, online and evaluation is effective	The ease and tone of verbal or chat room participation during the online CoP sessions	What participants report they *intend* to do with the information and the experience. Trust of participants to voice their experience and expertise equally
Qualitative results	Indication provided by positive email comments from participants after a session and in-between sessions	Low complexity: each session stood alone; participants were not “lost” if they did not participate in each session. Considered differing time zones of speakers/panelists as well as audience members	16 pre- and post-event planning meetings	Eight online CoP sessions implemented on topics such as exercising through the COVID-19 lockdown, mindfulness, cognition, gender, and team exercise for social participation	Persons living with HIV participants contributed with autopathographic testimonials. Participating clinicians described how the autopathographic material puts in perspective how they practice
Quantitative results	n/a	n/a	Managing the budget to cover costs of events and post-event tasks	48 out of 67 respondents reported connecting with someone related to the HIM CoP (72%) 19 short edited audiovisual presentations (in the library) 48+ h per up to four persons in preparation and implementation of CoP event	

### Measuring the goals and scope

Our assessment is that we widened the scope of what is often included in a CoP, what is accepted as authoritative. In scholarly work in health-related areas, especially in HIV/AIDS, we often circulate somber narratives regarding the most difficult aspects of living with HIV (stigmatization of the condition by others, physical and mental health problems, etc.). These may even have subtle but iatrogenic impacts (49). We did not gloss over the challenges, facts, and emotions stirred by living with HIV, but by highlighting the lived experience of people living with HIV, we emphasized on their resilience, astute coping mechanisms, living strategies (50), and even humor (a time-honored form of resistance in AIDS activism) (51). In our deliberations and in the qualitative evaluation responses, it was made clear that we were *interrupting the HIV somber narrative of scientific research* on HIV and included other possibilities such as autopathography, that is, patient-centered storytelling (52, 53), which include contrapuntal and “polyphonic narratives” (54). These are so because people living with HIV bring to bear a number of practical (social determinants of health) and philosophical aspects in their lives and how they uniquely weave them during treatment or simply living with a condition; they can also disagree with aspects of what is being discussed. In sum, we strived to be distinctive from stagnant, long, information-heavy, and victimhood narratives of scholarly presentations about HIV (and other medical conditions) firmly installed in the late 1980s (55).

### Measuring the context and structure

In this area, we boast of having retained the Ambassadors and a number of participants over the 2 years with their explicit satisfaction with the governance, leadership, and overall manner in which we carried out our activities. The CoP structure was an attempt to flatten, to some extent, the hierarchy of academic endeavors.

Flexibility of content and structure: Being flexible and making changes on the go was a productive approach. One example: when we noticed that the live online invited guest appearances (academic and community) were becoming long and stilted, we shortened the length of the presentation, provided the speakers with tips to “translate” scholarly material to media-friendly language and visuals, and offered to pre-record and edit 15- to 20-min sessions. This made the CoP sessions more agile and befitting of social media formats to be *consumed* synchronously and asynchronously. Thus, we followed trends in online structuration; sustained attention span, focus, concentration (56, 57); and, as importantly, we also included physical activities that would remind online participants of their bodies and avoid sedentarism and the many physical problems brought by sitting on online meetings (58). Including physical activity demonstrations online related to the theme of the event also helped reduce the anxiety of the physical demonstration presenters and afforded those online an unpressured choice to follow the exercise presented (59).

### Measuring the process and activities

Although attendance is not equivalent to engagement, we can ascertain through participant observation and the results of electronic evaluations that those who came to the online events were effectively engaged. One indicator of success is having retained all the participants logged in throughout each online session, having heard their verbal interventions, or having read their comments in the chat room. Participation in the chat room must be included as an indicator of engagement (60). Chat rooms offer space for those with disabilities, learning, engagement, and differing participation styles.

Attending and engaging: A total of 451 individuals participated live online. These are not unique instances. An average of three participants came back to several CoPs online over time. Across all eight sessions from October 2020 to September 2022, attendees included 173 persons living with HIV (32%), 116 persons affiliated to non-profit organizations (21.6%), 95 academic researchers (17.7%), and 67 clinical healthcare providers (12.5%), which included physicians, OTs, and PTs.

Connecting with others in the CoP: Across all eight CoP sessions, 48 out of 67 respondents (72%) reported connecting with someone related to the CoP. Some connections were brand new, and some were firmed up among colleagues who have collaborated before. Connections included participating in the online event preparation meetings, preparing poster or oral presentations for conferences, and connecting with other participants for purposes related to living with HIV and physical rehabilitation. These forms of participation range from transactional to peripheral (“apprentices”) and active (https://www.wenger-trayner.com/slide-forms-of-participation/; Accessed January 21, 2023).

### Measuring outcomes

Responses to the open-ended question on overall thoughts on the HIV in Motion Community of Practice elicited responses such as “I love the fact that you got people out of their chairs and exercising. The panel members were fantastic and looked at exercise from different perspectives.” Responses to the question “In what ways might you use the information shared at the HIV in Motion Community of Practice in your life or work?” elicited learnings and inspiration such as “I run a support group for folks living with HIV and I was hoping to get yoga incorporated but now I am definitely going to make sure to get this ball rolling.” It also prompted testimonials of clinical applications such as “[T]he info and live chats encouraged me to start working on our residence functional fitness program. So far, we had two sessions that included both the exercise portion as well as exploration of various topics such as exercise & mind-body-spirit connection, positive habit development, excuses for not exercising, and healthy snacking.”

Negative aspects to the evaluation did not outweigh the positive ones; they included technical problems (at their terminal) and wanting longer presentations and question and answer periods.

### Measuring impact

Autopathography: One indicator of impact we detected in the electronic evaluations and through participant observation was the trust of attending persons living with HIV to disclose and narrate powerful stories of physical activity and social participation. Participants living with HIV shared voluntarily and unscripted, on and off webcam, many autopathographic aspects of their lives and their accompanying reflections. For example, one 50+ person living with HIV told us that they had never spoken about their exercise experience in public and expressed they did not have much to offer but for years had climbed a well-known challenging mountain with a team. It was only through this reflection that they weighed their experiences in relation to health and social participation. Other instances were public and reported in our audiovisual library, including the lived experiences of persons living with HIV who have persevered and remained adaptable to challenges (e.g., by modifying and changing activities when they had become too straining due to changing co-morbidities). One example: A 50+ woman recounted humorously her struggle of leaving a wheelchair to start walking alone or with others. Tolerance for uncertainty and adaptability remain two of several sought-after attributes of patient empowerment (61).

The autopathographic narratives did not only originate from participants living with HIV. Video interviews with the YMCA coaches associated with the HIV in MOTION study for 2 years revealed struggles with physical and mental health challenges and how becoming coaches was a life-defining choice (https://hivinmotion.ca/tele-coaching-community-based-exercise-study). This attests to the sociological mechanisms of micro-recognition and mutual recognition (62) within the CoP. The impact of caregiving burnout among healthcare providers lost its significance after the year 2000 as effective HIV treatment became the proxy for quality of life with HIV and its normalization. The impact of conducting clinical and social–behavioral research on HIV also appears to be minimal, if it is reported at all.

Based on the literature, we infer that many participants, whether HIV+ or not, want to voice their experience, and when presented with respectful and equitable conditions, they take this opportunity. Beyond medical technical information and guidelines of practice, autopathography has the potential of helping us all understand the lived experience of patients and healthcare providers (52). This often unseen *impact* is an indicator of success in this area. In the evaluation form, one practitioner wrote “[this kind of online event] can help me share the ideas relating to physical activities that can easily be performed by people regardless of their fitness level or adapted for people that are using mobility aids [and] helps me promote recreational activities that are light or moderate in intensity as lifestyle physical activities that can easily be integrated in daily living that can, consequently, help person experience numerous health benefits.”

Mentorship: Including people living with HIV is a cornerstone of HIV community-based research. However, including emerging researchers and creating propitious conditions under which “patients” can truly collaborate with seasoned clinicians and established researchers is also reported in the literature as a desirable indicator of process (63, 64) Often, reported mentorship outcomes in communities of practice refer to close-knit healthcare provider settings (e.g., among early medical practitioners) and it has a teaching goal (28, 65, 66). Instead, our venture potentiated the *relational value* (67) of bringing together practitioners, patients, and academics. As in the case of working with people living with HIV, this type of mentorship required tact, care, and support. In the literature, this process of apprenticeship has been famously conceptualized by Lave and Wenger as “legitimate peripheral participation.” Researchers have applied the concept to the professional formation of health professionals (26).

In our electronic evaluation survey, we gathered opinions from students in the field who attended the synchronous sessions: “I am a therapeutic recreation student and will be doing a project in the coming weeks at the [HIV medical center]. I am here to learn about how to support this community and population alongside my studies.” One other student wrote, “[this type of] sessions can help practicum students understand various therapeutic approaches and research findings used across the globe; they can help my clients connect with our local recreation resources.”

## Discussion

HIV in MOTION was a multi-stakeholder community of practice, a form of integrated knowledge mobilization (KT) aimed to reduce the gaps between research evidence and collective. In this paper, we described the design, implementation, and evaluation of the CoP by applying a framework that included Goals and Scope, Context and Structure, Process and Activities, Outcomes, and Impact (see Table 1).

In terms of Context and Structure, one lesson learned is the need to have an Engagement Coordinator supporting the team as well as all those involved who are HIV+, which echoes what is being reported in the literature (68, 69). The tasks of the Engagement Coordinator are different from the academic administration of the study, often bringing in perspectives on how “patients” should receive the technical material or be explained technical language and processes and attending specifically to the concerns and ideas of people living with HIV.

In the area of Process and Activities, our outcomes reported earlier in this paper included the number of attendees, speakers, panelists, exercise demonstrators, as well as the number of times they repeatedly attended a CoP session. We spent at least 160+ h of our combined time in preparation meetings and activities, building our capacity to deliver online events and prepare the audiovisual library materials.

An important indicator of success around Process and Activities is having properly managed a modest fund to cover activities, honoraria, and costs of promotion and post-event editing. One related lesson learned from process and activities is to budget funds, researchers’ time, and effort generously. The iKT process is exhilarating but it constitutes a parallel effort for academics. In an academic environment where individuals with personal experience in health conditions are often compensated for their contributions to iKT, beyond just goodwill, academics should be ready to invest a considerable amount of effort into budget-related tasks. This might involve navigating through various stages within academic institutions to facilitate payments.

Health practitioners and researchers in this area have clamored that COVID-19 has shown that public and patient involvement is now necessary more than ever (70). When measuring *impact*, our reflection on the process elicits that the HIV in MOTION CoP contributed to challenging the echo chamber of HIV research meetings which favor the discourse of specialists, researchers, or clinicians by actively including the patient, clinicians, exercise staff, and students in the area. In the evaluation, one clinician wrote: “[This is] useful for my clinical practice. I work with clients who are HIV positive.” Functioning as an *anti-echo chamber* is a significant qualitative impact. The inclusion of diverse voices (gender, location, access to services, etc.) enhanced the conversation and brought in competing perspectives.

## Conclusion

The indicators of success isolated in this paper give an indication of the extent we meet the adapted criteria: Goals and Scope, Context and Structure, Process and Activities, Outcomes, and Impact.

For example, by designing the CoP for dialog across roles (patient, doctor, etc.), we increased the relational and intersectional values of the venture. CoPs with an inclusive goal of connecting personal and collective intersectionalities (e.g., physicians and AIDS activists both with high social capital in the field) can enact user-friendly “micro-processes of recognition and a breaking down of conventional hierarchies” (39). A micro-process can be as simple as a candid conversation between patients and doctors in a more power balanced setting outside the traditional patient–provider and medical history formats.

We realized how much effort goes into governance, design, implementation, after-event media editing, and approvals. At the time of this writing, we are on hiatus and looking for ways to transfer this modest but powerful legacy to a sister study; passing on this legacy has emerged as our greatest challenge so far.

In terms of structure, we managed well many technical aspects but could not completely avoid a degree of burnout. Putting together an ongoing online event requires a great deal of time, focus, and funds. However, one of our greatest limitations is not having been able yet to secure the sustainability of HIV in MOTION beyond the lifetime of the accompanying research study. Our gambit for sustainability is in having produced a slew of audiovisual materials that may be used in the development of HIV research teams, mentoring emerging clinicians, and to inform and inspire persons living with HIV.

Our recommendations based on these efforts and results is to continue fostering online instances of flexible dialogs that allow the voices of “patients,” clinicians, and academics to mix without apprehension that some should be more authoritative than others; it shows positive process, outcomes, and impact. There is a growing need and demand for democratic dialogical spaces for health practitioners, researchers, and patients such as the space offered by a community of practice. Also, we recommend easing our academic standards, which tend to be untenable for the lay person wanting to participate, to seek flexibility and being surprised. Communities of practice can be informal without diluting the informative, clinical, and scientific content and allowing the voice of patients to emerge but uninscribed in the authoritative business of academia.

## Data Availability

The data supporting the conclusions of this article will be made available by the authors, upon reasonable request to the corresponding authors.

## Footnotes

^1^“Lived experience” is defined in this context as a philosophical stance (one’s life well examined) and a phenomenological construct of the most indivisible form of experience (e.g., pain, stigma, happiness) (15).

^2^Process evaluations focus on the implementation logic while outcome evaluations focus on the results. In an evaluation, a process is “a way of talking about common action that cuts across program activities, observed interactions, and program content” (42–44).

## References

[1] Pyrko I Dörfler V Eden C. Thinking together: what makes communities of practice work? Hum Relat. (2016) 70(4):389–409. 10.1177/0018726716661040 28232754 PMC5305036

[2] Wenger E Wenger-Trayne B. Introduction to communities of practice. A brief overview of the concept and its uses (2015). Available at: https://www.wenger-trayner.com/introduction-to-communities-of-practice/ (Accessed 2022).

[3] Wenger E McDermott RA Snyder W. Cultivating communities of practice: A guide to managing knowledge. Boston, MA: Harvard Business Press (2002).

[4] Wenger ET. Communities of practice: learning as a social system. The Systems Thinker (2017). Available at: https://thesystemsthinker.com/communities-of-practice-learning-as-a-social-system/ (Accessed 2022).

[5] Hart A Davies C Aumann K Wenger E Aranda K Heaver B Mobilising knowledge in community–university partnerships: what does a community of practice approach contribute? Contemp Soc Sci. (2013) 8(3):278–91. 10.1080/21582041.2013.767470

[6] O'Brien KK Brown DA Corbett C Flanagan N Solomon P Vera JH AIDSImpact special issue—broadening the lens: recommendations from rehabilitation in chronic disease to advance healthy ageing with HIV. AIDS Care. (2020) 32(Suppl 2):65–73. 10.1080/09540121.2020.1739203 32208741

[7] O'Brien KK Tynan AM Nixon SA Glazier RH. Effectiveness of aerobic exercise for adults living with HIV: systematic review and meta-analysis using the Cochrane Collaboration Protocol. BMC Infect Dis. (2016) 16(179):182. 10.1186/s12879-016-1478-2 27112335 PMC4845358

[8] deBoer H Anddrews M Cudd S Leung E Petrie A Chan Carusone S Where and how does physical therapy fit? Integrating physical therapy into interprofessional HIV care. Disabil Rehabil. (2019) 41(15):1768–77. 10.1080/09638288.2018.1448469 29529881

[9] O'Brien KK Ibanez-Carrasco F Solomon P Harding R Brown D Ahluwalia P Research priorities for rehabilitation and aging with HIV: a framework from the Canada-international HIV and rehabilitation research collaborative (CIHRRC). AIDS Res Ther. (2020) 17(1):21. 10.1186/s12981-020-00280-5 32429973 PMC7236512

[10] Chetty L Cobbing S Chetty V. Physical activity and exercise for older people living with HIV: a scoping review. HIV/AIDS (Auckland). (2021) 13:1079–90. 10.2147/HIV.S336886 PMC8702781 34984030

[11] Simonik A Vader K Ellis D Kesbian D Leung P Jachyra P Are you ready? Exploring readiness to engage in exercise among people living with HIV and multimorbidity in Toronto, Canada: a qualitative study. BMJ Open. (2016) 6(3):e010029. 10.1136/bmjopen-2015-010029 26956163 PMC4785327

[12] Sahel-Gozin N Loutfy M O'Brien KK. Exploring experiences engaging in exercise from the perspectives of women living with HIV: a qualitative study. PLoS One. (2023) 18(6):e0286542. 10.1371/journal.pone.0286542 37267270 PMC10237415

[13] O'Brien KK Tynan AM Nixon SA Glazier RH. Effectiveness of progressive resistive exercise (PRE) in the context of HIV: systematic review and meta-analysis using the Cochrane Collaboration Protocol. BMC Infect Dis. (2017) 17(1):268. 10.1186/s12879-017-2342-8 28403830 PMC5389006

[14] Vancampfort D Mugisha J Richards J De Hert M Lazzarotto AR Schuch FB Dropout from physical activity interventions in people living with HIV: a systematic review and meta-analysis. AIDS Care. (2017) 29(5):636–43. 10.1080/09540121.2016.1248347 27794625

[15] Petitmengin C Remillieux A Valenzuela-Moguillansky C. Discovering the structures of lived experience. Phenomenol Cogn Sci. (2019) 18(4):691–730. 10.1007/s11097-018-9597-4

[16] Gunawardena CN Hermans MB Sanchez D Richmond C Bohley M Tuttle R. A theoretical framework for building online communities of practice with social networking tools. EMI Educ Media Int. (2009) 46(1):3–16. 10.1080/09523980802588626

[17] Jaldemark J. Theories of participation in online learning communities: an intersectional understanding. Int J Web Based Communities. (2012) 8(3):375–88. 10.1504/IJWBC.2012.048058

[18] Gagliardi AR Kothari A Graham ID. Research agenda for integrated knowledge translation (IKT) in healthcare: what we know and do not yet know. J Epidemiol Community Health. (2017) 71(2):105–6. 10.1136/jech-2016-207743 27647137 PMC5284465

[19] Kothari A Wathen CN. A critical second look at integrated knowledge translation. Health Policy (Amsterdam). (2012) 109(2):187–91. 10.1016/j.healthpol.2012.11.004 23228520

[20] Boland L Kothari A McCutcheon C Graham ID, and for the Integrated Knowledge Translation Research Network. Building an integrated knowledge translation (IKT) evidence base: colloquium proceedings and research direction. Health Res Policy Syst. (2020) 18(1):8. 10.1186/s12961-019-0521-3 31959184 PMC6972018

[21] Gainforth HL Hoekstra F McKay R McBride CB Sweet SN Martin Ginis KA Integrated knowledge translation guiding principles for conducting and disseminating spinal cord injury research in partnership. Arch Phys Med Rehabil. (2021) 102(4):656–63. 10.1016/j.apmr.2020.09.393 33129763

[22] Struminger B Arora S Zalud-Cerrato S Lowrance D Ellerbrock T. Building virtual communities of practice for health. Lancet. (2017) 390(10095):632–4. 10.1016/S0140-6736(17)31666-5 28816126 PMC6402556

[23] Simeonov D Kobayashi K Grenier A. How to implement an integrated knowledge mobilization approach. Cham: Springer International Publishing (2020). p. 351–9.

[24] Zhang W Watts S. Online communities as communities of practice a case study. J Knowl Manag. (2008) 12(4):55–71. 10.1108/13673270810884255

[25] McKellar KA. Evaluating extra-organizational communities of practice. Ann Arbor: University of Toronto (Canada) (2019). p. 157.

[26] Orsmond P McMillan H Zvauya R. It’s how we practice that matters: professional identity formation and legitimate peripheral participation in medical students: a qualitative study. BMC Med Educ. (2022) 22(1):91. 10.1186/s12909-022-03107-1 35139839 PMC8830078

[27] Jiménez-Zarco I González-González I Saigí-Rubió F Torrent-Sellens J. The co-learning process in healthcare professionals: assessing user satisfaction in virtual communities of practice. Comput Human Behav. (2015) 51:1303–13. 10.1016/j.chb.2014.11.057

[28] Gallagher DM Hirschhorn LR Lorenz LS Piya P. Developing a community of practice for HIV care: supporting knowledge translation in a regional training initiative. J Contin Educ Health Prof. (2017) 37(1):27–36. 10.1097/CEH.0000000000000141 28212116

[29] Symon B Spurr J Brazil V. Simulcast: a case study in the establishment of a virtual community of simulation practice. Adv Simul. (2020) 5(1):5. 10.1186/s41077-020-00122-4 PMC7251887 32514383

[30] Kulandaivelu Y. Examining the development of a community of practice in paediatric project ECHO®. Ann Arbor: University of Toronto (Canada) (2019). p. 113.

[31] Woods A Cashin A Horstmanshof L. The social construction of nurse educator professional identities: exploring the impact of a community of practice through participatory action research. J Adv Nurs. (2022) 78(8):2522–36. 10.1111/jan.15200 35384031 PMC9540668

[32] Bermejo-Caja CJ Koatz D Orrego C Perestelo-Pérez L González-González AI Ballester M Acceptability and feasibility of a virtual community of practice to primary care professionals regarding patient empowerment: a qualitative pilot study. BMC Health Serv Res. (2019) 19(1):403. 10.1186/s12913-019-4185-z 31221215 PMC6587268

[33] Abma TA Cook T Rämgård M Kleba E Harris J Wallerstein N. Social impact of participatory health research: collaborative non-linear processes of knowledge mobilization. Educ Action Res. (2017) 25(4):489. 10.1080/09650792.2017.1329092 30135617 PMC6101044

[34] Ibáñez-Carrasco F Worthington C Rourke S Hastings C. Universities without walls: a blended delivery approach to training the next generation of HIV researchers in Canada. Int J Environ Res Public Health. (2020) 17(12):4265. 10.3390/ijerph17124265 32549263 PMC7344852

[35] Eaton AD Ibáñez-Carrasco F Craig SL Chan Carusone S Montess M Wells GA A blended learning curriculum for training peer researchers to conduct community-based participatory research. Action Learn Res Pract. (2018) 15(2):139–50. 10.1080/14767333.2018.1462143

[36] Ibáñez-Carrasco F Watson JR Tavares J. Supporting peer researchers: recommendations from our lived experience/expertise in community-based research in Canada. Harm Reduct J. (2019) 16(1):55–55. 10.1186/s12954-019-0322-6 31481067 PMC6724244

[37] McKellar KA Berta W Cockerill R Cole DC Saint-Charles J. Application of an evaluation framework for extra-organizational communities of practice: assessment and refinement. Can J Program Eval. (2020) 35(2):149–69. 10.3138/cjpe.69796

[38] Murad A Hyde N Chang S Lederman R Bosua R Pirotta M Quantifying use of a health virtual community of practice for general practitioners’ continuing professional development: a novel methodology and pilot evaluation. J Med Internet Res. (2019) 21(11):e14545. 10.2196/14545 31774401 PMC6906624

[39] Stephansen HC Couldry N. Understanding micro-processes of community building and mutual learning on twitter: a ‘small data’ approach. Inf Commun Soc. (2014) 17(10):1212–27. 10.1080/1369118X.2014.902984

[40] Alcalde-Rabanal JE Becerril-Montekio VM Langlois EV. Evaluation of communities of practice performance developing implementation research to enhance maternal health decision-making in Mexico and Nicaragua. Implement Sci. (2018) 13(1):41. 10.1186/s13012-018-0735-8 29530055 PMC5848447

[41] Moore JL Bjørkli C Havdahl RT Lømo LL Midthaug M Skjuve M A qualitative study exploring contributors to the success of a community of practice in rehabilitation. BMC Med Educ. (2021) 21(1):282. 10.1186/s12909-021-02711-x 34001073 PMC8130156

[42] Patton MQ. Developmental evaluation: applying complexity concepts to enhance innovation and use. New York: Guilford Press (2011).

[43] Fridrich A Jenny G Bauer G. The context, process, and outcome evaluation model for organisational health interventions. BioMed Res Int. (2015) 2015:12. 10.1155/2015/414832 PMC4628757 26557665

[44] W.K. Kellogg Foundation. The step-by-step guide to EVALUATION. How to become savvy evaluation consumers (2017) 1–264.

[45] Smith MG Witte M Rocha S Basner M. Effectiveness of incentives and follow-up on increasing survey response rates and participation in field studies. BMC Med Res Methodol. (2019) 19(1):230. 10.1186/s12874-019-0868-8 31805869 PMC6896692

[46] Miller RE. Reference communities: applying the community of practice concept to development of reference knowledge. Public Serv Q. (2011) 7(1–2):18–26. 10.1080/15228959.2011.572772

[47] West J. Authentic voices: utilising audio and video within an online virtual community. Soc Work Educ. (2008) 27(6):665–70. 10.1080/02615470802201762

[48] Lau B Sharma I Manku S Kobylianski J Wong LY Ibáñez-Carrasco F Considerations for developing and implementing an online community-based exercise intervention with adults living with HIV: a qualitative study. BMJ Open. (2022) 12(4):e059294. 10.1136/bmjopen-2021-059294 35428647 PMC9014056

[49] Ibanez-Carrasco F. Feeling sick, looking cured!: the Iatrogenic effects of HIV public health policy on HIV-positive gay men. In: Hindmarch S Orsini M Gagnon M, editors. Seeing red: HIV/AIDS and public policy in Canada. Toronto: University of Toronto Press (2018). p. 102–22. 10.3138/9781487510305-006

[50] Solomon P O'Brien KK McGuff R Sankey M. Living strategies for disability in men ageing with HIV in Ontario, Canada: a longitudinal qualitative study. BMJ Open. (2019) 9:e031262. 10.1136/bmjopen-2019-031262 31481379 PMC6731853

[51] De Moor K. Diseased pariahs and difficult patients: humour and sick role subversions in queer HIV/AIDS narratives. Cult Stud. (2005) 19(6):737–54. 10.1080/09502380500365705

[52] Aronson JK. Autopathography: the patient’s tale. Br Med J. (2000) 321(7276):1599–602. 10.1136/bmj.321.7276.1599 11124195 PMC1119270

[53] Tembeck T. Selfies of ill health: online autopathographic photography and the dramaturgy of the everyday. Soc Media Soc. (2016) 2(1):2056305116641343. 10.1177/2056305116641343

[54] Ezzy D. Illness narratives: time, hope and HIV. Soc Sci Med. (2000) 50(5):605–17. 10.1016/S0277-9536(99)00306-8 10658842

[55] Munt SR. Argumentum ad misericordiam: the cultural politics of victim media. Fem Media Stud. (2017) 17(5):866–83. 10.1080/14680777.2016.1259176

[56] Webster JG. The marketplace of attention: how audiences take shape in a digital age. Cambridge: The MIT Press (2014).

[57] MacPherson PR. Blended teaching: a guide for applying flexible practices during COVID-19. Hamilton, ON: McMaster University (2020).

[58] Singh J. Are video calls hurting your employees? 80% of workers have physical ailments from online meetings. Employee Benefit News (Online) (2022). Available at: https://www.benefitnews.com/news/are-video-calls-hurting-your-employees-80-of-workers-have-physical-ailments-from-online-meetings (Accessed September 24, 2023).

[59] Browne Jeffrey C Liu M. Replacing live video of a meeting participant with recorded video of the meeting participant during an online meeting (2018).

[60] Sarkar A Rintel S. The rise of parallel chat in online meetings: how can we make the most of it? Microsoft Research Blog (2021). Available at: https://www.microsoft.com/en-us/research/blog/the-rise-of-parallel-chat-in-online-meetings-how-can-we-make-the-most-of-it/. (Access September 24, 2023).

[61] Strout TD Hillen M Gutheil C Anderson E Hutchinson R Ward H Tolerance of uncertainty: a systematic review of health and healthcare-related outcomes. Patient Educ Couns. (2018) 101(9):1518–37. 10.1016/j.pec.2018.03.030 29655876

[62] Jacobs EA Kohrman C Lemon M Vickers DL. Teaching physicians-in-training to address racial disparities in health: a hospital-community partnership. Public Health Reports (1974). (2003) 118(4):349–56. 10.1016/S0033-3549(04)50260-1 PMC1497548 12815083

[63] Hemming D Phinney J. Mentoring library school interns at a distance: insights gained from a remote community of practice. Le Mentorat à Distance des Stagiaires en Bibliothéconomie. (2021) 16(2):1–6. 10.21083/partnership.v16i2.6654

[64] Steinert Y. Faculty development: from workshops to communities of practice. Med Teach. (2010) 32(5):425–8. 10.3109/01421591003677897 20423263

[65] Obura T Brant WE Miller F Parboosingh IJ. Participating in a community of learners enhances resident perceptions of learning in an e-mentoring program: proof of concept. BMC Med Educ. (2011) 11(1):3. 10.1186/1472-6920-11-3 21266070 PMC3041783

[66] McDonald PW Viehbeck S. From evidence-based practice making to practice-based evidence making: creating communities of (research) and practice. Health Promot Pract. (2007) 8(2):140–4. 10.1177/1524839906298494 17384405

[67] Faraj S von Krogh G Monteiro E Lakhani KR. Special section introduction—online community as space for knowledge flows. Inf Syst Res. (2016) 27(4):668–84. 10.1287/isre.2016.0682

[68] Duong D. Five lessons from a year of virtual patient partnerships. Can Med Assoc J. (2021) 193(29):E1145–6. 10.1503/cmaj.1095953 34312173 PMC8321302

[69] Kipping S Stuckey MI Hernandez A Nguyen T Riahi S. A web-based patient portal for mental health care: benefits evaluation. J Med Internet Res. (2016) 18(11):2. 10.2196/jmir.6483 PMC5131190 27852556

[70] Murphy E Tierney E Ní Shé E Killilea M Donaghey C Daly A COVID-19: public and patient involvement, now more than ever. HRB Open Res. (2020) 3:35. 10.12688/hrbopenres.13067.1 32666039 PMC7327728

