# Supplementary material for: HIV in MOTION: a community of practice on physical rehabilitation for and by people living with HIV and their allies
Source: Front Rehabil Sci. 2023 Oct 6;4:1154692. doi: 10.3389/fresc.2023.1154692 (PMC10588699; doi:10.3389/fresc.2023.1154692)
Supplement: Supplementary file 1 [file Image1.pdf]

Supplemental File 1: CoP HIM evaluation questions.

1. Demographic information: Affiliation (Person living with HIV, Researcher, Clinician / Health Care Provider, Student, Non-profit organization or community-based organization or HIV / AIDS service organization Trainee (any level), Fitness personnel, Government agency staff, age, gender, and Indigenous identity.
2. Overall, how would you rate this HIV in MOTION event? (1 lowest to 5 highest)
3. Please rate on the scale from 1 (low level of knowledge) to 10 (high level of knowledge) your understanding/knowledge of "living with HIV, exercise, and fitness trackers" before the HIV in MOTION session of [date].
4. Please rate on the scale from 1 (low level of knowledge) to 10 (high level of knowledge) your understanding/knowledge of "living with HIV, exercise, and fitness trackers" after the HIV in MOTION session of [date].
5. Please indicate what best describes your level of agreement on the following statement: “I will consider incorporating the skills and knowledge learned during this session in my current projects and/or in future initiatives” (a 5-point Likert scale from “strongly agree” to “strongly disagree”)
6. What were your overall thoughts on the HIV in Motion Community of Practice Session?
7. In what ways will you use the information shared at the HIV in Motion Community of Practice in your life or work?
8. Did you have a chance to CONNECT with someone related to the HIV in MOTION Community of Practice, before, during or after the event?
9. If you have attended HIV in MOTION before, have you had the chance to consult or use materials from our digital library or recommend them to others? Please check as many options as you need to. Here is the link to the library http://bit.ly/HIVinMOTIONsite. It contains materials from studies on physical exercise and living with HIV, edited recordings of live events, and other eLearning materials.
10. Will you be using the materials contained in the HIV in MOTION website/digital library?
11. What suggestions do you have for future sessions with the HIV in Motion Community of Practice? Your suggestions may be about content, guests, or format.
